# Dietary Pineapple Pomace Complex Improves Growth Performance and Reduces Fecal Odor in Weaned Piglets by Modulating Fecal Microbiota, SCFAs, and Indoles

**DOI:** 10.3390/ani15243600

**Published:** 2025-12-15

**Authors:** Shengnan Yu, Jiahao Jin, Minglin Zheng, Fuquan Yin, Wenchao Liu, Zhihui Zhao, Liyuan Wang, Yuxia Chen

**Affiliations:** 1 College of Coastal Agriculture Science, Guangdong Ocean University, Zhanjiang 524088, China; 19856932930@163.com (S.Y.); 13512757245@163.com (J.J.); 18312132904@163.com (M.Z.); yinfuquan01@163.com (F.Y.); liuwc@gdou.edu.cn (W.L.); zhzhao@gdou.edu.cn (Z.Z.); 2 Shenzhen Branch, Guangdong Laboratory for Lingnan Modern Agriculture, Agricultural Genomics Institute at Shenzhen, Chinese Academy of Agricultural Sciences, Shenzhen 518124, China; wangliyuan01@caas.cn

**Keywords:** pineapple pomace complex, weaned piglets, growth performance, fecal microbiota, short-chain fatty acids, skatole

## Abstract

This study aimed to investigate the effects of pineapple pomace as a functional feed additive on growth performance, fecal microbiota composition, short-chain fatty acids (SCFAs), and odor substance emissions in weaned piglets. The experiment utilized 90 weaned three-way crossbred piglets (Duroc–Yorkshire–Guoshou Black Pig) at 28 days of age, randomly divided into three groups of 10 pigs each: the control group (CON, basal diet), the fish meal group (FM, basal diet + 2% imported fish meal), and the pineapple pomace group (PPC, basal diet + 2% pineapple pomace compound). The results indicated the PPC group exhibited significantly higher average daily gain (ADG) (*p* < 0.05) and significantly lower feed conversion ratio (FCR) (*p* < 0.05) than the CON group. 16S rRNA sequencing analysis revealed that the PPC group significantly increased fecal microbiota species richness and diversity (*p* < 0.01) while enriching beneficial bacteria including *Lactobacillus*, *Bifidobacterium*, and butyrate-producing bacteria such as *Blautia*. Concurrently, acetic acid and butyric acid levels in feces were significantly elevated in the PPC group (*p* < 0.05), with butyric acid production markedly higher than in the FM group (*p* < 0.05). However, no significant differences were observed in the levels of branched-chain fatty acids (BCFAs), such as isobutyric acid and isovaleric acid (*p* < 0.05). Moreover, PPC supplementation led to a significant decrease in fecal levels of indole and 3-methylindole (skatole) (*p* < 0.05). In summary, dietary inclusion of 2% pineapple pomace improves growth performance and intestinal health in weaned piglets by modulating the fecal microbiota, promoting beneficial SCFA production, and inhibiting protein putrefaction. These findings support PPC as an effective and sustainable alternative to fish meal, contributing to the development of environmentally friendly feeding strategies in pig production.

## Simple Summary

The discovery of sustainable ingredients to replace expensive fish meal and reduce farming pollution is a key goal in pig production. This study investigated the potential of pineapple pomace, a fiber-rich fruit byproduct, as a functional feed additive for weaned piglets. We found that adding just 2% pineapple pomace to the diet significantly improved the piglets’ growth rate and feed efficiency. It also made positive changes in the gut by increasing the diversity of microbes, boosting beneficial bacteria like *Lactobacillus*, and raising levels of health-promoting short-chain fatty acids. Furthermore, it effectively reduced the production of foul-smelling compounds in the manure. In conclusion, pineapple pomace is a promising, eco-friendly alternative to fish meal that enhances piglet growth and gut health while lessening environmental impact.

## 1. Introduction

Pineapple (*Ananas comosus*), a widely cultivated tropical fruit, generates substantial pomace as a byproduct of its processing industry. This pomace is rich in dietary fiber, vitamins, and minerals, endowing it with great potential as a functional feed ingredient [1,2]. Moreover, pineapple pomace contains a spectrum of bioactive compounds with demonstrated physiological effects. These include bromelain, a proteolytic enzyme complex known to enhance protein digestibility and exert anti-inflammatory activity; diverse polyphenols such as catechins that exhibit potent antioxidant capacity; and functional polysaccharides with immunomodulatory and gut-protective properties [3,4,5]. Collectively, these components contribute to multiple documented bioactivities, including modulation of gut microbiota, enhancement of intestinal barrier function, and mitigation of oxidative stress and inflammation. The annual production of this byproduct in major growing regions like Zhanjiang reaches hundreds of thousands of tons, and its underutilization poses both a waste of resources and an environmental burden. Concurrently, the growing emphasis on environmental sustainability and health in animal husbandry has spurred interest in developing novel, green feed additives.

Dietary fiber is known to enhance animal production performance and gut health by modulating the gut microbiota and promoting the production of short-chain fatty acids (SCFAs) [6]. For instance, Liu et al. reported that dietary alfalfa meal altered the hindgut microbial community, increased SCFA concentrations, and reduced the incidence of diarrhea in piglets [7]. This is particularly relevant for weaned piglets, which face severe physiological stress and gut microbiota dysbiosis during the transition from a milk-based to a solid diet. Consequently, the post-weaning period represents a critical window for nutritional intervention to stabilize the gut ecosystem and mitigate the risk of diarrhea. As a rich source of natural fiber, pineapple pomace has shown preliminary promise in improving animal growth and immunity [4,8]. However, systematic research on its effects in weaned piglets—particularly concerning nutrient utilization, the gut microbiota, and key metabolites like SCFAs and odorous compounds—remains limited. This research gap stems from three fundamental factors: the traditional research paradigm that prioritizes direct nutritional substitution over functional modulation; the historical lack of standardized processing protocols for agricultural by-products like pineapple pomace compared to established ingredients like fish meal; and the methodological complexity required to evaluate multi-component ingredients through integrated analyses of gut microbiome and metabolomic profiles.

Moreover, direct comparative studies between pineapple pomace supplementation and traditional protein sources like fish meal are notably scarce, yet hold significant scientific and practical value. Although fish meal is widely used due to its high protein content and excellent amino acid profile, it faces challenges such as price volatility, limited resource sustainability, and primarily nutritional functions. In contrast, pineapple pomace not only represents an economically viable alternative but also serves as a functional ingredient with prebiotic properties. Our study therefore addresses this critical gap by systematically evaluating PPC as a multi-functional alternative to fish meal, representing a shift from the traditional “substitution” paradigm toward a “functional modulation” approach that investigates how different nutritional components work in concert to regulate gut health and overall production performance.

Therefore, we hypothesized that pineapple pomace complex (PPC) could act as a prebiotic to optimize the gut microbiota in weaned piglets. This modulation would promote beneficial SCFA production, suppress protein putrefaction and the generation of odorous compounds, and ultimately improve host growth performance and intestinal health. To test this, the present study was designed to systematically evaluate the effects of dietary PPC supplementation on growth performance, fecal microbiota composition, SCFA levels, and emissions of odor compounds (indole and skatole) in weaned piglets, with comparisons made against fish meal supplementation. The findings aim to provide a theoretical foundation and practical guidance for the utilization of pineapple pomace as a partial fish meal alternative in environmentally friendly feed formulations.

## 2. Materials and Methods

### 2.1. Animals, Diet and Experimental Design

The experimental protocol applied in this study followed the guidelines of the Animal Care and Use Committee of Guangdong Ocean University (authorization number GDOU-20240820-03, 20 August 2024).

The pineapple pomace complex (PPC) used in this study was provided by Zhanjiang Boshan Jiuqian Biotechnology Co., Ltd. (Zhanjiang, China), while the piglets were sourced from Guangxi Pig Treasure Agriculture and Animal Husbandry Co., Ltd. (Nanning, China). Ninety (45 male, 45 female) 28-day-old weaned three-way crossbred piglets (Duroc–Yorkshire–Guoshou Black Pig, average weight 5.39 ± 0.09 kg) were selected for a 21-day trial. Environmental temperature was maintained at 25–28 °C, and humidity at 60–70% throughout the trial. The 28-day-old weaned piglets were randomly divided into 3 groups and randomly assigned to one of three dietary groups. Each diet group comprised 3 replicate pens containing ten pigs per pen (balanced across sex; five males and three females per pen), with pig and diet assignment to pens performed using a random number generator. The control group (CON) was fed a basal diet, while the other two groups were fed the same diet supplemented with the fish meal at 2% (FM group) and the pineapple pomace complex at 2% (PPC group; the PPC inclusion level was selected based on optimal growth performance observed in preliminary trials conducted by a specialized feed manufacturer). Although the specific fermentation process and final formulation are proprietary, the manufacturer provided a complete nutrient composition analysis report of the raw pineapple pomace, which has been included as Supplementary Materials (see Supplementary Materials S1) and confirms its core compositional parameters. All pigs had free access to water and were fed ad libitum daily. All pigs were weighed on days 1 and 21 post-weaning to monitor production performance, and pen-level feed intake was monitored to calculate average daily gain (ADG), average daily feed intake (ADFI), and feed conversion ratio (FCR). The basal diet (Table 1) was formulated to meet or exceed the nutritional requirements for weaned piglets as recommended by the U.S. NRC (2012) [9].

### 2.2. Growth Performance Assessment and Fecal Sample Collection and Processing

Body weight was determined on day 1 and 21, and the feed intake was recorded daily. The average daily gain (ADG), average daily feed intake (ADFI), and feed conversion ratio (FCR) for the entire trial period (1–21 days) were also calculated.

On day 21, fresh fecal samples were collected from six randomly selected piglets per replicate. Approximately 50 g of feces was collected from each piglet, taking care to avoid contamination with bedding or urine. Samples were immediately placed into sterile centrifuge tubes. For microbial community analysis, samples were rapidly frozen in liquid nitrogen and subsequently stored at −80 °C within one hour. For the analysis of short-chain fatty acids (SCFAs), indole, and skatole, samples were treated with 1% thymol as a preservative, homogenized by vortexing, and then stored at −80 °C until analysis.

### 2.3. 16S rRNA Sequencing and Bioinformatics Analysis

Total genomic DNA samples were extracted using the OMEGA Soil DNA Kit (M5635-02) (Omega Bio-Tek, Norcross, GA, USA), following the manufacturer’s instructions, and stored at −20 °C prior to further analysis. The quantity and quality of extracted DNAs were measured using a NanoDrop NC2000 spectrophotometer (Thermo Fisher Scientific, Waltham, MA, USA) and agarose gel electrophoresis, respectively.

PCR amplification of the bacterial 16S rRNA genes V3–V4 region was performed using the forward primer 338F (5′-ACTCCTACGGGAGGCAGCA-3′) and the reverse primer 806R (5′-GGACTACHVGGGTWTCTAAT-3′). Sample-specific 7 bp barcodes were incorporated into the primers for multiplex sequencing. The PCR components contained 5 μL of buffer (5×), 0.25 μL of Fast pfu DNA Polymerase (5U/μL), 2 μL (2.5 mM) of dNTPs, 1 μL (10 uM) of each Forward and Reverse primer, 1 μL of DNA Template, and 14.75 μL of ddH2O. Thermal cycling consisted of initial denaturation at 98 °C for 5 min, followed by 25 cycles consisting of denaturation at 98 °C for 30 s, annealing at 53 °C for 30 s, and extension at 72 °C for 45 s, with a final extension of 5 min at 72 °C. PCR amplicons were purified with Vazyme VAHTSTM DNA Clean Beads (Vazyme, Nanjing, China) and quantified using the Quant-iT PicoGreen dsDNA Assay Kit (Invitrogen, Carlsbad, CA, USA). After the individual quantification step, amplicons were pooled in equal amounts, and pair-end 2–250 bp sequencing was performed using the Illumina NovaSeq platform with NovaSeq 6000 SP Reagent Kit (500 cycles) at Suzhou PANOMIX Biomedical Tech Co., Ltd. (Suzhou, China).

Raw sequencing data (Raw Reads) were processed using QIIME2 software (version 2022.11): First, low-quality reads (Q-value < 20, length < 150 bp) and adapter sequences were removed to obtain high-quality reads (Clean Reads). Tags were assembled through overlapping regions, and after chimera sequence removal, effective tags (Effective Tags) were obtained. Effective Tags were clustered into operational taxonomic units (OTUs) based on 97% sequence similarity and annotated with species information by aligning against the Silva 138 database.

Based on OTU results, α-diversity indices were calculated: Chao1 index (species richness), Shannon index (species diversity), and Simpson index (species evenness). One-way ANOVA was used to compare differences between groups. Beta diversity was analyzed via principal coordinate analysis (PCoA, based on Bray–Curtis distance) to visually illustrate gut microbiota structural differences among groups. Linear discriminant analysis (LDA) combined with effect size measurement (LEfSe, LDA score > 3) was employed to identify significantly different microbial communities between groups. The Pearson correlation coefficient between fecal microbiota and SCFAs was calculated using the Ouyi Cloud platform (https://cloud.oebiotech.com/, accessed on 23 August 2025), and a heatmap was generated.

### 2.4. Analysis of Short-Chain Fatty Acids in Feces

Around 50 mg of feces per sample was accurately weighed into sterile 1.5 mL microcentrifuge tubes (n = 6). Subsequently, samples were homogenated for 1 min with 500 μL of water and 100 mg of glass beads, and then centrifuged at 4 °C for 10 min at 12,000 rpm. 200 μL supernatant was extracted with 100 μL of 15% phosphoric acid and 20 μL of 375 μg/mL of 4-methylvaleric acid solution as internal standard (IS) and 280 μL ether. Subsequently, the samples were centrifuged at 4 °C for 10 min at 12,000 rpm after vortexing for 1 min and the supernatant was transferred into the vial prior to Gas Chromatograph System for VFA determination (TRACE 1300, Thermo, Waltham, MA, USA).

### 2.5. Analysis of Indoleacetic Acid and Skatole in Feces

All the analytical standards were prepared in 50% methanol at the concentration of 1000 µg/mL as stock solution. The working solutions were diluted serially with 10% methanol. All stock solutions and working standard solutions were stored at −20 °C.

The fecal samples were weighted and homogenized in 2 mL centrifuge tube. Specifically, 100 μL of 80% methanol was added, and the mixture was vortexed for 60 s. Subsequently, two steel balls were added, and the tubes were placed into a tissue grinder. Grinding was performed at 55 Hz for 1 min, and this grinding step was repeated at least once. Then, 900 μL of 10% methanol was added, followed by another round of grinding at 55 Hz for 1 min; this entire procedure was repeated at least twice. The samples were centrifuged at 12,000 rpm and 4 °C for 5 min. An aliquot of the supernatant was diluted five-fold with 10% methanol. For analysis, 100 μL of this diluent was combined with 100 μL of a 20 ppb Trp-d5 internal standard solution, vortexed for 30 s, and 150 μL of the final mixture was transferred to an LC-MS vial for injection.

### 2.6. Statistical Analysis

All experimental data were first organized using Excel 2019, then statistically analyzed with SPSS 26.0 software. Growth performance, SCFAs, indole, and skatole content data were analyzed using one-way analysis of variance (ANOVA), with Duncan’s multiple range test for post hoc comparisons between groups. Microbial α-diversity indices were assessed using the Kruskal–Wallis H test. All results are expressed as “mean ± standard error of the mean (Mean ± SEM)”. Differences were considered statistically significant at *p* < 0.05 and highly statistically significant at *p* < 0.01.

## 3. Results

### 3.1. The Effects of PPC on Growth Performance of Weaned Piglets Growth Performance

As shown in Table 2, the inclusion of PPC in the diet significantly improved the growth performance of weaned piglets. No significant differences in initial body weight (BW) were observed among the groups (*p* > 0.05). At the end of the trial, final BW showed no significant differences, although numerical increases were noted in both the FM and PPC groups compared to the CON group (*p* > 0.05). However, significant differences were observed in average daily gain (ADG) and feed conversion ratio (FCR). Throughout the entire trial period, the PPC group had the highest ADG, which was significantly higher than that of the CON group (*p* < 0.05). The FM group showed an intermediate ADG that was not statistically different from either the CON or PPC groups. Similarly, for FCR, the PPC group exhibited the highest efficiency, significantly outperforming than the CON group (*p* < 0.05), while the FM group again fell between the two groups. In contrast, no significant differences in ADFI were observed among the groups (*p* > 0.05). In conclusion, dietary supplementing with 2% pineapple pomace complex significantly enhanced the growth rate of weaned piglets by improving feed conversion efficiency, demonstrating superior effects compared to supplementing with 2% fish meal alone.

### 3.2. The Effects of PPC on the Fecal Microbiota Characterization of Weaned Piglets Fecal Microbiota Community Characerization

**Figure 1:**
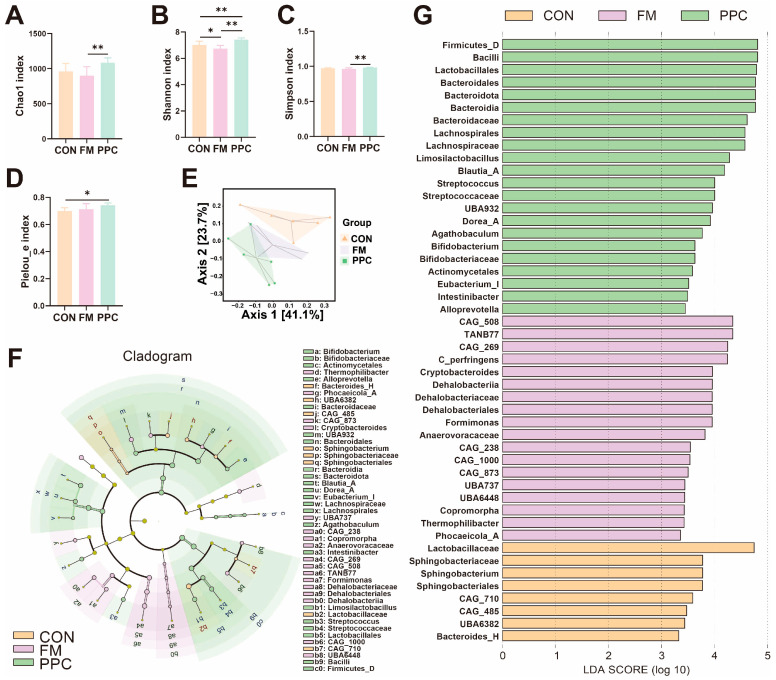
Effects of PPC on the Fecal Microbiota Characterization of Weaned Piglets: Analysis of alpha diversity in fecal microbiota across groups using the (**A**) Chao1 index, (**B**) Shannon index, (**C**) Simpson index, and (**D**) Pielou_e index. ** indicates extremely significant differences (*p* < 0.01), while * indicates significant differences (*p* < 0.05). (**E**) Beta diversity analysis (Principal Coordinate Analysis, PCoA), (**F**) Cladogram and (**G**) LDA distribution and LefSe analysis identifying species with significant intergroup differences (LDA scores > 3). CON, basal diet; FM, basal diet + 2% imported fish meal; PPC, basal diet + 2% pineapple pomace complex (n = 6).

Different dietary treatments significantly influenced the diversity and composition of the intestinal microbiota in weaned piglets. Regarding α-diversity, the PPC group demonstrated significantly higher Chao1 richness index and Shannon diversity index compared to the FM group (*p* < 0.05), but no significant difference was observed relative to the CON group (*p* > 0.05) (Figure 1A–D). This suggests that pineapple pomace supplementation altered microbial richness and evenness, with effects distinct from fish meal supplementation.

Regarding beta diversity, as illustrated by principal coordinate analysis (PCoA), clear structural separation among these three groups was revealed (Figure 1E). The first two principal coordinates (PC1 and PC2) explained 41.1% and 23.7% of the total variance, respectively. Notably, the PPC group formed a distinct cluster that was clearly separated from both the CON and FM groups, suggesting that dietary pineapple pomace induced a unique shift in the gut microbial community structure.

Further phylogenetic cladogram and LDA effect size (LefSe) analyses identified group-specific microbial clusters (Figure 1F,G). The CON group was characterized by clusters within the phylum *Bacteroidota*, such as *UBA6382* and *Bacteroides_H*. In contrast, the FM group showed significant enrichment of bacteria associated with protein metabolism, including multiple genera from the *Bacillota* phylum (e.g., *Clostridium_perfringens*, *TANB77*, and members of the *Peptostreptococcales order*), alongside *Formimonas* and *Cryptobacteroides*, suggesting a community shift toward protein fermentation and anaerobic respiration. The PPC group, however, demonstrated a profound remodeling of the gut microbiota, selectively enriching taxa involved in carbohydrate fermentation and probiotic functions. The overall abundance of the *Bacillota phylum* was exceptionally high. Notably, we observed a marked increase in *lactobacilli*, including the order *Lactobacillales*, the family *Lactobacillaceae*, and the genus *Limosilactobacillus*. Crucially, several short-chain fatty acid (SCFA)-producing bacteria, particularly butyrate producers, were significantly enriched. These included *Blautia_A*, *Dorea_A*, *Eubacterium_I*, and *Agathobaculum*. The abundances of *Bifidobacterium* and *Streptococcus* were also significantly elevated compared to the other groups. In summary, while dietary pineapple pomace did not increase overall microbial richness, it specifically reshaped the community structure by enriching key functional groups—especially carbohydrate-fermenting and probiotic bacteria—which may represent a key mechanism underpinning the improved host growth performance.

### 3.3. The Effects of PPC on SCFAs in Weaned Piglets SCFA Characerization and Correlation Analysis

**Figure 2:**
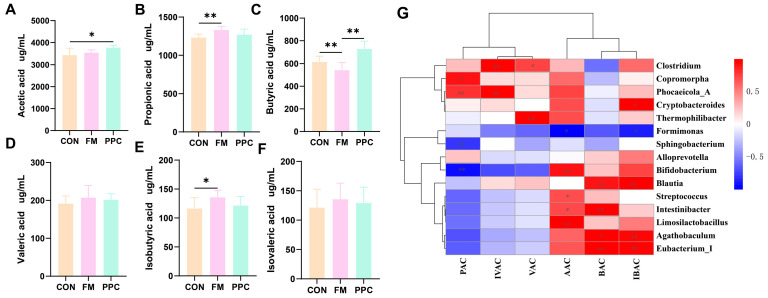
Effects of PPC on SCFAs in Weaned Piglets: (**A**) Acetic acid, (**B**) Propionic acid (**C**) Butyric acid, (**D**) Valeric acid, (**E**) Isobutyric acid, and (**F**) Isovaleric acid. Black dots represent individual sample sizes within each group (n = 6). (**G**) Pearson correlation analysis between intestinal flora and SCFAs: (AAC) acetic acid, (PAC) propionic acid, (BAC) butyric acid, (VAC) valeric acid, (BAC) isobutyric acid, and (IVAC) isovaleric acid. * indicates *p* < 0.05, ** indicates *p* < 0.01. CON, basal diet; FM, basal diet + 2% imported fish meal; PPC, basal diet + 2% pineapple pomace complex (n = 6).

Dietary supplementation with pineapple pomace complex (PPC) significantly modulated the composition and concentration of SCFAs in the feces of weaned piglets. As shown in Figure 2, the concentrations of acetic acid, and butyric acid in the feces of the PPC group were significantly elevated compared to the CON group (*p* < 0.05, Figure 2C). Notably, butyrate production in the PPC group was also significantly higher than in the FM group, indicating pineapple pomace ’s superior efficacy in stimulating butyrate production. Conversely, the isobutyric acid concentration in the FM group was significantly higher than the CON group (*p* < 0.05, Figure 2E), while no significant difference was observed between the PPC and CON groups (*p* > 0.05). In summary, pineapple pomace enhanced overall SCFA production, specifically promoting acetate and butyrate, while demonstrating a unique advantage over fish meal in boosting butyrate production.

Pearson correlation analysis between the top 30 abundant fecal microbiota and microbial metabolites further elucidated the microbiological mechanism by which pineapple pomace modulates microbial metabolism in weaned piglets (Figure 2G). The significantly elevated concentrations of acetate and butyrate in the PPC group were strongly positively correlated with the enrichment of specific beneficial bacteria. These included *Limosilactobacillus* (a lactic acid producer), *Bifidobacterium* (an acetate producer), and key butyrate-producing genera such as *Blautia* and *Rectobacillus*. Conversely, the concentrations of *isobutyrate* and isovalerate—branched-chain fatty acids indicative of protein putrefaction—were significantly negatively correlated with beneficial communities and/or positively correlated with bacteria like *Clostridium* and *Phocaeicola_A*. In summary, dietary pineapple pomace optimizes the intestinal SCFA profile by selectively enriching beneficial bacteria involved in carbohydrate fermentation (e.g., *Limosilactobacillus*, *Blautia*) while suppressing microbial pathways linked to protein putrefaction. This shift in the gut microbial ecology represents a key mechanism through which pineapple pomace improves intestinal health.

### 3.4. Fecal Odorous Compounds Analysis

**Figure 3:**
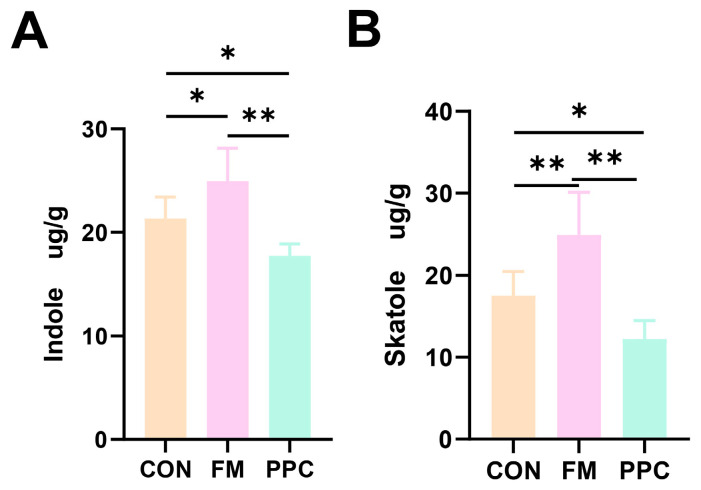
Effects of PPC on indole and skatole in weaned piglets (**A**) Indole, (**B**) Skatole. * indicates *p* < 0.05, ** indicates *p* < 0.01. CON, basal diet; FM, basal diet + 2% imported fish meal; PPC, basal diet + 2% pineapple pomace complex (n = 6).

Dietary intervention significantly influenced the levels of indole-type odor compounds in the feces of weaned piglets. As shown in Figure 3, the PPC group exhibited a pronounced reduction in the concentrations of both indole (IND) and 3-methylindole (skatole, 3-MI) compared to the CON and FM groups. Specifically, the PPC group showed a highly significant decrease in IND compared to the FM group (*p* < 0.01) and a significant decrease compared to the CON group (*p* < 0.05). Similarly, the concentration of 3-MI in the PPC group was significantly lower than in the CON group (*p* < 0.05) and highly significantly lower than in the FM group (*p* < 0.01). These results demonstrate that pineapple pomace supplementation effectively mitigates the production of key fecal odor compounds, with an efficacy superior to that of fish meal.

## 4. Discussion

### 4.1. The Effects of PPC on Growth Performance of Weaned Piglets

In the present study, dietary supplementation with PPC significantly increased the average daily gain (ADG) and reduced the feed conversion ratio (FCR) in weaned piglets, demonstrating effects that surpassed those of the fish meal (FM) group. The absence of significant differences in average daily feed intake (ADFI) among all groups indicates that the enhanced growth performance stemmed not from increased consumption, but from improved nutrient utilization efficiency, likely mediated by optimized intestinal function.

This improvement can be attributed to the distinctive chemical composition of the pineapple pomace complex used in this trial (for detailed composition see Supplementary Materials S1). The material was characterized by high total dietary fiber content (56.19%), which aligns with previous reports on pineapple pomace [2,4]. While the soluble fiber fraction was not quantitatively determined in our analysis, the known physicochemical properties of pineapple pomace fiber provide mechanistic insights. The growth-promoting effect of fiber is often linked to its physicochemical properties. While some high-fiber diets can reduce feed intake by promoting satiety or through physical gut fill [10], the particular fiber profile in PPC appears to be beneficial. Literature indicates that pineapple pomace contains both soluble and insoluble fiber components, with soluble fibers like pectin being particularly fermentable by gut microbiota [11]. The specific fiber composition in PPC appears to be beneficial. For instance, insoluble non-starch polysaccharides have been shown to increase ADFI by shortening the mean retention time in the gastrointestinal tract, whereas soluble fibers can prolong retention time and increase satiety [10]. The fermentable nature of PPC’s fiber fraction likely supported microbial activity in the hindgut, leading to the production of short-chain fatty acids (SCFAs) that provide an additional energy source for the host and improve gut health, thereby contributing to the enhanced nutrient utilization and growth performance observed. Our results align with findings that certain fiber sources, like wheat bran, can improve feed conversion efficiency in weaned piglets [11]. Furthermore, the performance benefits observed with PPC are consistent with other functional supplements. For instance, silybin has been shown to improve growth performance by lowering FCR through enhanced intestinal health and antioxidant capacity [12]. This parallel suggests that PPC may exert its benefits through a similar multi-faceted mechanism involving both gut microbiota modulation and potential systemic effects.

Notably, although the FM group received a high-protein supplement, its growth performance was inferior to the PPC group. This discrepancy may be attributed to undigested protein fermenting in the hindgut. Putrefactive bacteria, such as Clostridium, can decompose excess protein into harmful metabolites like ammonia, biogenic amines, and hydrogen sulfide, which compromise intestinal epithelial integrity and can lead to diarrhea risk in weaned piglets [13,14]. This mechanism underscores a key advantage of PPC: its efficacy is primarily driven by its high content of functional fiber, which supports a healthy gut ecosystem by serving as a fermentable substrate for beneficial microbiota. While pineapple pomace is known to contain other bioactive compounds, their specific contribution in the present study requires further verification. This highlights the practical value of pineapple pomace as a multi-functional and sustainable strategy to reduce reliance on expensive imported fish meal in livestock production.

### 4.2. The Effects of PPC on the Fecal Microbiota Characterization of Weaned Piglets

Our 16S rRNA sequencing results demonstrated that dietary supplementation with PPC significantly reshaped the fecal microbial ecosystem in weaned piglets. This remodeling is closely linked to the high dietary fiber content of PPC, with its fiber components acting as prebiotics that selectively promoted the proliferation of carbohydrate-fermenting bacteria. Specifically, PPC enriched beneficial genera involved in carbohydrate fermentation, such as *Lactobacillus*, *Blautia*, and *Bifidobacterium*, while suppressing putative potential protein spoilage bacteria like *Clostridium*. The significant enrichment of *Lactobacillus* and *Bifidobacterium* aligns with previous reports on fiber-rich and polyphenol-containing ingredients. For instance, pineapple by-products have been shown to preferentially stimulate the growth of these beneficial taxa [15]. Similarly, other functional fibers, such as resistant starch, are known to promote fecal *lactobacilli* and *bifidobacteria* in pigs [16]. These carbohydrate-utilizing bacteria, selectively stimulated by PPC fiber, are key microorganisms responsible for fiber degradation and short-chain fatty acid (SCFA) production. The proliferation of these carbohydrate-utilizing bacteria is crucial, as they drive the fermentation of dietary fiber into SCFAs, thereby improving host lipid metabolism and overall gut health [17]. Our findings confirm that pineapple pomace acts as an effective prebiotic substrate, fostering a gut environment conducive to beneficial fermentation.

In stark contrast, the FM group exhibited an enrichment of microorganisms associated with protein degradation and anaerobic respiration, such as *Clostridium perfringens* and members of the *Peptostreptococcaceae* family. This pattern is a typical microbial response to the fermentation of undigested dietary protein in the hindgut [18]. The distinct microbial ecologies induced by PPC (carbohydrate fermentation) and FM (protein fermentation) underscore a fundamental difference in their functional impacts on gut health. The PPC-induced microbiota, characterized by a higher *Firmicutes*-to-*Bacteroidota* (F/B) ratio and a robust community of SCFA producers, is more aligned with intestinal homeostasis and improved growth performance [19], thereby providing a microbiological basis for the superior growth outcomes observed in the PPC group.

### 4.3. The Effects of PPC on SCFAs in Weaned Piglets

Our study found that supplementing diets with 2% pineapple pulp complex significantly increased acetate, and butyrate concentrations in the feces of weaned piglets, whereas the levels of propionate, valerate, and branched-chain fatty acids (BCFAs) were not significantly affected. Notably, PPC exhibited a superior butyrate-promoting effect compared to fish meal.

The elevated SCFA production can be directly attributed to the fermentation of dietary fiber from pineapple pomace by the fecal microbiota. This is consistent with the high total dietary fiber content of the PPC used in this study (Supplementary Materials S1), which served as a fermentable prebiotic substrate. This aligns with studies on other fibrous ingredients, such as alfalfa meal and cereal brans, which similarly increase cecal or fecal SCFA levels [7,20]. Dietary fiber functions as a bioactive compound that alleviates gut dysbiosis and normalizes the intestinal environment by modulating microbial composition [6,21]. Our 16S rRNA analysis confirms that this prebiotic effect selectively stimulated beneficial bacteria proficient in fiber degradation, such as *Bifidobacterium* and *Blautia*, which are established SCFA producers. The physiological benefits of this SCFA enrichment are multifaceted. SCFAs, particularly butyrate, serve as the primary energy source for colonocytes, crucially maintaining intestinal barrier function by upregulating mucin and antimicrobial peptide secretion, regulating cell proliferation and apoptosis [22,23,24]. Furthermore, SCFAs lower gut pH, inhibiting the colonization of enteric pathogens [7]. Acetate, as the most abundant SCFA, acts as a key signaling molecule in host metabolism and an energy substrate for peripheral tissues [6,17]. Conversely, the FM group showed a significant increase in branched-chain fatty acids (BCFAs), such as iso-butyrate. This finding is critical because BCFAs are established hallmark metabolites of protein putrefaction [25]. In contrast, the PPC group exhibited a relative reduction in these compounds. This contrast indicates that PPC supplementation shifts microbial fermentation in the hindgut away from proteolysis and toward more beneficial carbohydrate fermentation. Consequently, this dietary shift reduces the production of harmful protein-derived metabolites.

This shift in fermentation patterns is mechanistically explained by our Pearson correlation analysis, which revealed strong positive correlations between acetate and *Bifidobacterium*, and between butyrate and key producers like *Blautia_A* and *Agathobaculum*. These correlations are consistent with previous reports linking these bacterial genera to SCFA production [6,15] and provide a direct link between the PPC-induced microbial changes and the observed metabolic outcomes. In conclusion, pineapple pomace alters the gut environment by enriching specific SCFA-producing bacteria, which in turn modifies the metabolic profile to enhance beneficial fatty acids and suppress harmful protein fermentation products.

### 4.4. Effects of PPC on Fecal Odor Compound and Indole Levels in Weaned Piglets

A key finding of this study was the significant reduction in fecal indole (IND) and 3-methylindole (skatole) levels in the PPC group compared to both the CON and FM groups. This reduction represents a significant benefit for optimizing the farming environment. Skatole, an aromatic compound derived from the microbial putrefaction of tryptophan in the feces, is a primary contributor to odor in swine barns and can accumulate in adipose tissue, leading to “boar taint” and diminished pork quality, thereby causing economic losses [26,27].

The mechanism by which pineapple pomace reduces these odorous compounds is likely twofold. Firstly, dietary composition is a primary determinant of skatole production. The PPC diet had a lower crude protein content than the FM diet, which may have reduced the amount of undigested protein reaching the hindgut. Furthermore, the fermentable dietary fiber in PPC (crude fiber: 3.88% vs. 3.18% in CON) promotes carbohydrate-fermenting bacteria over proteolytic species, thereby shifting microbial metabolism away from tryptophan degradation into skatole [28,29]. This finding establishes a clear causal pathway: PPC-mediated modulation of the gut microbiota—characterized by the promotion of saccharolytic bacteria and concurrent suppression of proteolytic taxa (as demonstrated in our microbial community analysis)—directly attenuates the proteolytic fermentation of tryptophan into indole and skatole (as quantitatively confirmed by our metabolomic assays), thereby mechanistically explaining the significant reduction in fecal odor intensity. Secondly, the *Lactobacillus* species enriched in the PPC group may directly inhibit indole-producing bacteria, such as *Escherichia coli* and *Clostridium*, through the secretion of bacteriocins and by lowering gut pH, further curtailing the production of these malodorous compounds [30,31].

In summary, the inclusion of pineapple pomace in the diet of weaned piglets enhances growth performance through a synergistic mechanism. The fermentable fiber promotes a healthy fecal microbiota, which in turn improves host health and nutrient utilization. This is achieved by: (1) enriching beneficial bacteria and generating SCFAs that enhance intestinal barrier function, regulate immunity, and provide energy, thereby improving nutrient digestion and absorption [22,23,32,33]; and (2) simultaneously suppressing proteolytic bacteria and the production of harmful metabolites like skatole and indole, which benefits both piglet health and the environment. This study underscores the value of pineapple pomace as a functional feed ingredient for sustainable swine production.

## 5. Conclusions

In conclusion, dietary supplementation with 2% pineapple pomace complex (PPC) serves as a functional prebiotic that effectively remodels the fecal microbiota in weaned piglets. By enriching beneficial taxa, including *Lactobacillus*, *Bifidobacterium*, and key butyrate producers, PPC enhances the production of SCFAs (notably butyrate) and suppresses proteolytic fermentation. These microbial improvements translate into enhanced growth performance and feed efficiency, alongside a significant reduction in fecal odorous compounds such as skatole and indole. The overall efficacy of PPC was superior or equivalent to 2% imported fish meal. Therefore, as a low-cost and readily available agricultural byproduct, pineapple pomace represents a high-value alternative to fish meal. Its adoption not only helps lower feed costs and import dependency but also provides a strategic approach for fostering greener and more sustainable swine production.

## Figures and Tables

**Table 1:** The composition and nutrient levels of experimental diets.

Project	CON	FM	PPC
Ingredient, %			
Corn	61.00	59.00	59.50
Soybean Meal	12.00	9.80	10.10
Expanded Soybeans	10.00	10.00	10.00
Fish Meal	4.00	6.00	4.00
Soybean Oil	3.00	3.00	3.00
Whey Powder	3.00	3.00	3.00
Salt	0.40	0.40	0.40
Limestone Powder	0.70	0.70	0.70
Dicalcium Phosphate	0.80	0.80	0.80
Lysine Hydrochloride	0.90	0.90	0.90
Methionine	0.10	0.10	0.10
Threonine	0.10	0.10	0.10
Premix ^1^	4.00	4.00	4.00
Pineapple Pomace Complex	0.00	0.00	2.00
Corn Starch	0.00	2.20	1.40
Total	100.00	100.00	100.00
**Nutritional Level**			
Dry Matter, %	88.64	88.28	87.87
Digestible Energy, MJ/kg	14.21	14.35	14.18
Crude Fiber, %	3.18	3.74	3.88
Crude Protein, %	18.33	20.05	18.52

^1^ The premix provided the following per kilogram of complete diet: Vitamin A, 8000 IU; Vitamin D_3_, 2000 IU; Vitamin E, 60IU; Vitamin K_3_, 1 mg; Vitamin B1, 1.5 mg; Vitamin B2, 4.0 mg; Vitamin B6, 2 mg; Vitamin B12, 0.04 mg; Biotin, 0.2 mg; Folic acid, 1 mg; Nicotinic acid, 20 mg; Pantothenic acid, 10 mg; choline, 400 mg; Cu, 150 mg; Fe, 100 mg; Zn, 100 mg; Mn, 40 mg; I, 0.5 mg; Se, 0.2 mg.

**Table 2:** The effects of dietary supplementation with pineapple pomace complex (PPC) on growth performance of weaned piglets.

Item ^1^	CON	FM	PPC	SEM ^2^	*p*-Value
BW of day 1 (kg)	5.35	5.35	5.34	0.09	0.799
BW of day 21 (kg)	10.52	10.81	10.83	0.12	0.574
ADG (g/d)	247 ^b^	257 ^ab^	261 ^a^	2.67	0.046
ADFI (g/d)	340	343	349	2.05	0.156
FCR	1.38 ^a^	1.35 ^ab^	1.34 ^b^	0.01	0.032

^1^ CON, basal diet; FM, basal diet + 2% imported fish meal; PPC, basal diet + 2% pineapple pomace complex. BW, body weight; ADG, average daily gain; ADFI, average daily feed intake; FCR, feed conversion rate. ^2^ SEM, standard error of the mean. ^a, b^ Values in the same row with different letters are significantly different (*p* < 0.05). Results are presented as mean ± SEM (n = 6).

## Data Availability

The data analyzed in this study are available from the corresponding author upon request.

## Supplementary Materials

The following supporting information can be downloaded at: https://www.mdpi.com/article/10.3390/ani15243600/s1, Pineapple Pomace (BXB-1)—Reference Nutritional Value of Preserved Pineapple Pomace Feed Ingredient.

## References

[1] Nath P.C., Ojha A., Debnath S., Neetu K., Bardhan S., Mitra P., Sharma M., Sridhar K., Nayak P.K. (2023). Recent Advances in Valorization of Pineapple (*Ananas comosus*) Processing Waste and by-Products: A Step towards Circular Bioeconomy. Trends Food Sci. Technol..

[2] Chaudhary S., Singh B. (2024). Pineapple By-Products Utilization: Progress towards the Circular Economy. Food Humanit..

[3] Alves Nobre T., de Sousa A.A., Pereira I.C., Carvalho Pedrosa-Santos Á.M., Lopes L.D.O., Debia N., El-Nashar H.A., El-Shazly M., Islam M.T., Castro e Sousa J.M.D. (2024). Bromelain as a natural anti-inflammatory drug: A systematic review. Nat. Prod. Res..

[4] Jose M., Himashree P., Sengar A.S., Sunil C.K. (2022). Valorization of food industry by-product (pineapple pomace): A study to evaluate its effect on physicochemical and textural properties of developed cookies. Meas. Food.

[5] Hadidi M., Ibarz A., Khaksar F.B., Hasiri Z. (2020). Polysaccharides from pineapple core as a canning by-product: Extraction optimization, chemical structure, antioxidant and functional properties. Int. J. Biol. Macromol..

[6] Hu R., Li S., Diao H., Huang C., Yan J., Wei X., Zhou M., He P., Wang T., Fu H. (2023). The Interaction between Dietary Fiber and Gut Microbiota, and Its Effect on Pig Intestinal Health. Front. Immunol..

[7] Liu B., Wang W., Zhu X., Sun X., Xiao J., Li D., Cui Y., Wang C., Shi Y. (2018). Response of Gut Microbiota to Dietary Fiber and Metabolic Interaction with SCFAs in Piglets. Front. Microbiol..

[8] Selani M.M., Brazaca S.G.C., Dos Santos Dias C.T., Ratnayake W.S., Flores R.A., Bianchini A. (2014). Characterisation and Potential Application of Pineapple Pomace in an Extruded Product for Fibre Enhancement. Food Chem..

[9] National Research Council (NRC) (2012). Nutrient Requirements of Swine: Eleventh Revised Edition.

[10] Vasconcelos T.S., Thomaz M.C., Castelini F.R., Alvarenga P.V.A., De Oliveira J.A., Ramos G.F., Ono R.K., Milani N.C., Dos Santos Ruiz U. (2020). Evaluation of Pineapple Byproduct at Increasing Levels in Heavy Finishing Pigs Feeding. Anim. Feed Sci. Technol..

[11] Xu Y., Zhou C., Lu Y., Guo X., Zong M., Zhu J., Zhou P., Pang J., Peng X., Sun Z. (2025). Multi-Omic Analysis for Dietary Supplementation of Different Ratios of Soluble and Insoluble Fiber on Intestinal Microbiota, Metabolites and Inflammation of Weaned Piglets. J. Integr. Agric..

[12] Cai L., Gao G., Yin C., Bai R., Li Y., Sun W., Pi Y., Jiang X., Li X. (2023). The Effects of Dietary Silybin Supplementation on the Growth Performance and Regulation of Intestinal Oxidative Injury and Microflora Dysbiosis in Weaned Piglets. Antioxidants.

[13] Erinle T.J., De Oliveira M.J.K., Htoo J.K., Mendoza S.M., Columbus D.A. (2025). Effect of Indigestible Dietary Protein on Growth Performance and Health Status of Weaned Pigs. J. Anim. Sci..

[14] Gao J., Yin J., Xu K., Li T., Yin Y. (2019). What Is the Impact of Diet on Nutritional Diarrhea Associated with Gut Microbiota in Weaning Piglets: A System Review. BioMed Res. Int..

[15] Campos D.A., Coscueta E.R., Vilas-Boas A.A., Silva S., Teixeira J.A., Pastrana L.M., Pintado M.M. (2020). Impact of Functional Flours from Pineapple By-Products on Human Intestinal Microbiota. J. Funct. Foods.

[16] Metzler-Zebeli B.U., Canibe N., Montagne L., Freire J., Bosi P., Prates J.A.M., Tanghe S., Trevisi P. (2019). Resistant Starch Reduces Large Intestinal pH and Promotes Fecal *Lactobacilli* and *Bifidobacteria* in Pigs. Animal.

[17] Chong C.W., Liew M.S., Ooi W., Jamil H., Lim A., Hooi S.L., Tay C.S.C., Tan G. (2024). Effect of Green Banana and Pineapple Fibre Powder Consumption on Host Gut Microbiome. Front. Nutr..

[18] Li Y., Han Y., Zhao Q., Tang C., Zhang J., Qin Y. (2022). Fermented Soy and Fish Protein Dietary Sources Shape Ileal and Colonic Microbiota, Improving Nutrient Digestibility and Host Health in a Piglet Model. Front. Microbiol..

[19] Liu R., He J., Ji X., Zheng W., Yao W. (2021). A Moderate Reduction of Dietary Crude Protein Provide Comparable Growth Performance and Improve Metabolism via Changing Intestinal Microbiota in Sushan Nursery Pigs. Animals.

[20] Zhao J., Liu P., Wu Y., Guo P., Liu L., Ma N., Levesque C., Chen Y., Zhao J., Zhang J. (2018). Dietary Fiber Increases Butyrate-Producing Bacteria and Improves the Growth Performance of Weaned Piglets. J. Agric. Food Chem..

[21] Xu G., Huang J., Chen W., Zhao A., Pan J., Yu F. (2024). The Influence of Increasing Roughage Content in the Diet on the Growth Performance and Intestinal Flora of Jinwu and Duroc × Landrace × Yorkshire Pigs. Animals.

[22] Liu A., Wang B., Wang M., Tang R., Xu W., Xiao W. (2025). L-Theanine Alleviates Ulcerative Colitis by Repairing the Intestinal Barrier through Regulating the Gut Microbiota and Associated Short-Chain Fatty Acids. Food Chem. Toxicol..

[23] Han X., Ma Y., Ding S., Fang J., Liu G. (2023). Regulation of Dietary Fiber on Intestinal Microorganisms and Its Effects on Animal Health. Anim. Nutr..

[24] Forsan C.F., Ávila P.F., Masarin F., Lopes P.R.M., Brienzo M. (2025). Cellooligosaccharides and Xylooligosaccharides: Production Processes, Potential Prebiotics, and Metabolism Routes by *Lactobacillus* and *Bifidobacterium*. Food Res. Int..

[25] Yin Y., Cai J., Zhou L., Xing L., Zhang W. (2022). Dietary Oxidized Beef Protein Alters Gut Microbiota and Induces Colonic Inflammatory Damage in C57BL/6 Mice. Front. Nutr..

[26] Lin Y., Deng D., Ma X., Yu M., Lu Y., Song M., Jiang Q. (2025). Advances in Production and invitro Degradation of Skatole in Livestock and Poultry. Chin. J. Anim. Sci. Vet. Med..

[27] Li Y., Liu Y., Mu C., Zhang C., Yu M., Tian Z., Deng D., Ma X. (2024). Magnolol-Driven Microbiota Modulation Elicits Changes in Tryptophan Metabolism Resulting in Reduced Skatole Formation in Pigs. J. Hazard. Mater..

[28] Zhu X., Zhang Y., Yang G., Liu H. (2021). Research Progress on Production Mechanism and Emission Reduction of Skatole in Livestock and Poultry. Chin. J. Anim. Nutr..

[29] Li X., Jensen B.B., Canibe N. (2019). The Mode of Action of Chicory Roots on Skatole Production in Entire Male Pigs Is Neither via Reducing the Population of Skatole-Producing Bacteria nor via Increased Butyrate Production in the Hindgut. Appl. Environ. Microbiol..

[30] Anjana, Tiwari S.K. (2022). Bacteriocin-Producing Probiotic Lactic Acid Bacteria in Controlling Dysbiosis of the Gut Microbiota. Front. Cell. Infect. Microbiol..

[31] Zhang D., Ji H., Wang S., Liu Y., Chen M., Liu H. (2023). Lactobacillus-Driven Feed Fermentation Regulates Microbiota Metabolism and Reduces Odor Emission from the Feces of Pigs. mSystems.

[32] Qiu Y., Tang J., Wang L., Yang X., Jiang Z. (2024). Fermented Corn–Soybean Meal Improved Growth Performance and Reduced Diarrhea Incidence by Modulating Intestinal Barrier Function and Gut Microbiota in Weaned Piglets. Int. J. Mol. Sci..

[33] Dong S., Zhang N., Wang J., Cao Y., Johnston L.J., Ma Y. (2025). Effects of Medium- and Short-Chain Fatty Acids on Growth Performance, Nutrient Digestibility, Gut Microbiota and Immune Function in Weaned Piglets. Animals.

