# Supplementary material for: Dietary Pineapple Pomace Complex Improves Growth Performance and Reduces Fecal Odor in Weaned Piglets by Modulating Fecal Microbiota, SCFAs, and Indoles
Source: Animals (Basel). 2025 Dec 15;15(24):3600. doi: 10.3390/ani15243600 (PMC12729931; doi:10.3390/ani15243600)
Supplement: Supplementary file 1 [file animals-15-03600-s001.zip › Supplementary Material S1.pdf]

Pineapple Pomace (BXB-1)—

Reference Nutritional Value of Preserved Pineapple Pomace Feed Ingredient

Item	As Sampled	Dry Matter Basis
Moisture/%	83.7	
Dry Matter/%	16.3	
Crude Protein (CP)/%	1.58	9.68
Acid Detergent Insoluble Crude Protein (ADICP)/%	0.2	1.2
Neutral Detergent Insoluble Crude Protein (NDICP)/%	0.3	1.54
Soluble Protein as % of Crude Protein (SP%CP)	/	52
Rumen Degradable Protein as % of Crude Protein (RDP/CP%)	/	82
Acid Detergent Fiber (ADF)/%	6.89	42.25
Neutral Detergent Fiber (aNDF)/%	9.16	56.19
Ash-Free Neutral Detergent Fiber (aNDF)/%	8.95	54.89
Lignin/%	1.17	7.2
Non-Fiber Carbohydrates (NFC)/%	3.60	22.08
Starch/%	1.58	9.7
Water Soluble Carbohydrates (WSC)/%	0.24	1.5
Ethanol Soluble Carbohydrates (ESC)/%	0.03	0.2
Fat/%	0.39	2.38
Ash/%	1.0	3.83
Total Digestible Nutrients (TDN)/%	8.48	52
Net Energy for Lactation (NEL)/(Mcal/kg)	0.18	1.09
Net Energy for Maintenance (NEM)/(Mcal/kg)	0.16	0.97
Net Energy for Gain (NEG)/(Mcal/kg)	0.07	0.43
Relative Feed Value (RFV)	/	93
Relative Forage Quality (RFQ 48h)	/	53
Calcium/%	0.13	0.8
Phosphorus/%	0.02	0.15
Magnesium/%	0.03	0.19
Potassium/%	0.27	1.63
Sulfur/%	0	0.03
Chloride Ion/%	0.06	0.37
30-hour In Vitro True Dry Matter Digestibility (IVTD)/%	/	59
30-hour Neutral Detergent Fiber Digestibility (NDFD)/%	/	27
Neutral Detergent Fiber Digestion Rate/(kd_hr)	/	1.74
Milk Production per Ton of Dry Matter/(kg/ton)	/	850
Methionine/%	/	0.13

Lysine/%	/	0.34
Horse Digestible Energy/(Mcal/Kg)	/	2.02
Acid Soluble Protein/%	0.62	2.37
Acid Soluble Protein as % of Crude Protein	/	24.5
Crude Fiber/%	4.12	15.77
Total Acid	1.24	4.75
